# Effective Photodynamic Therapy for Colon Cancer Cells Using Chlorin e6 Coated Hyaluronic Acid-Based Carbon Nanotubes

**DOI:** 10.3390/ijms21134745

**Published:** 2020-07-03

**Authors:** Prabhavathi Sundaram, Heidi Abrahamse

**Affiliations:** Laser Research Centre, Faculty of Health Sciences, University of Johannesburg, Johannesburg 2028, South Africa; prabhavathis@uj.ac.za

**Keywords:** colon cancer, chlorin e6, carbon nanotubes, hyaluronic acid, photodynamic therapy, photosensitizers

## Abstract

Colon cancer is the third major cancer contributor to mortality worldwide. Nanosized particles have attracted attention due to their possible contribution towards cancer treatment and diagnosis. Photodynamic therapy (PDT) is a cancer therapeutic modality that involves a light source, a photosensitizer and reactive oxygen species. Carbon nanotubes are fascinating nanocarriers for drug delivery, cancer diagnosis and numerous potential applications due to their unique physicochemical properties. In this study, single walled carbon nanotubes (SWCNTs) were coupled with hyaluronic acid (HA) and chlorin e6 (Ce6) coated on the walls of SWCNTs. The newly synthesized nanobiocomposite was characterized using ultraviolet-visible spectroscopy, Fourier transform electron microscopy (FTIR), X-ray diffraction analysis (XRD), particle size analysis and zeta potential. The loading efficiency of the SWCNTs-HA for Ce6 was calculated. The toxicity of the nanobiocomposite was tested on colon cancer cells using PDT at a fluence of 5 J/cm^2^ and 10 J/cm^2^. After 24 h, cellular changes were observed via microscopy, LDH cytotoxicity assay and cell death induction using annexin propidium iodide. The results showed that the newly synthesized nanobiocomposite enhanced the ability of PDT to be a photosensitizer carrier and induced cell death in colon cancer cells.

## 1. Introduction

Cancer is a deadly disease characterised by the formation and growth of abnormal cells that proliferate uncontrollably [1]. The specific causes of cancer include obesity, infections and UV radiation as reported by the WHO. Colorectal cancer (CRC) is the third most commonly diagnosed cancer in males and females, and if CRC metastasizes to other parts of the body, the 5-year survival rate of patients is 15% [2]. Current treatments for cancer are radiotherapy, chemotherapy, surgical resection, and immunotherapy. Currently, available treatments have severe side effects like poor bioavailability and non-specificity, killing healthy cells. Researchers are optimistic about the potential of nanomaterials to target cancer cells and increase the bioavailability of the drug [3].

Photodynamic therapy (PDT) is a relatively novel cancer treatment, which involves a light source, photosensitizer (PS) and reactive oxygen species. When the PS molecule is irradiated at a wavelength, it induces production and activation of singlet oxygen leading to cell necrosis or apoptosis [4]. Chlorin e6 (Ce6) is a naturally derived chlorophyll-a derivative, also termed photochlorin. It emerges as a prospective PS for PDT, with the main advantages of Ce6 being its low toxicity, easy synthesis and production, fast and selective accumulation in the target tissue, strong absorption wavelength at 650–670 nm, high singlet oxygen quantum yield, and almost no side effects to the skin [5,6]. However, the solubility of PS or drugs plays major roles in PDT-related cancer treatment [7]. The major issues associated with the solubility was prominently enhanced by nanotechnology using different nanocarrier systems such as polymeric nanoparticles [8], liposomes [9], micelles [10], carbon nanotubes (single-walled and multi-walled) [11] and metal-based nanoparticles [12] that have been designed.

Carbon nanotubes (CNTs) are rolled up by graphene sheets so that both ends are open forming hollow cylindrical structures. Based on the layer formation, CNTs are named as single-walled carbon nanotubes (SWCNTs) and multi-walled carbon nanotubes (MWCNTs), each showing a variation in properties. SWCNTs possess unique physicochemical properties that improve their quality as nanocarriers and have a wide variety of applications [13]. SWCNTs often enter the cells by direct penetration but MWCNTs enter via the endocytosis pathway [14]. SWCNTs improve the loading of drugs, DNA, proteins, antibodies, polymers [15] and photodrugs [11] and facilitates both covalent and non-covalent functionalization of targeting moieties that make them unique nano carriers in drug delivery systems [16]. Notably, the carboxylated SWCNTs show excellent biocompatibility in cancer therapy [17]. It has been reported that the SWCNT_S_ and PS showed an increased cytotoxic effect in MCF-7 cells and the combination therapy proved to be an effective alternative therapy for the successful treatment of breast cancer with reduced side-effects [18].

In this study, we synthesized a nanobiocomposite consisting of hyaluronic acid-coupled SWCNTs coated with Ce6 for efficient PDT of colon cancer cells. We successfully prepared the nanobiocomposite, which was characterised using different physicochemical characterisation methods. The maximum loading efficiency of SWCNTs for the strong non-covalent bonding π-π interaction of Ce6 was calculated. The dispersibility of the SWCNT was improved after the addition of biopolymer hyaluronic acid on the delivery system. It showed excellent anticancer activity against Caco-2 cells compared to free Ce6.

## 2. Results

### 2.1. UV-Vis Spectroscopy Study

**Figure 1:**
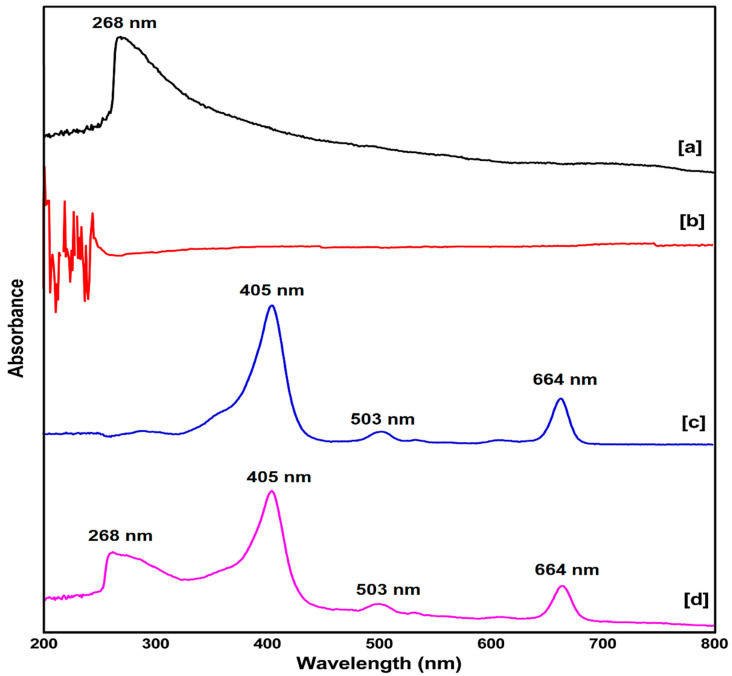
UV-vis spectroscopy of (**a**) SWCNTs (**b**) H.A (**c**) Ce6 (**d**) SWCNTs-HA-Ce6.

The primary confirmation of successful synthesis of the nanobiocomposite was done using the absorbance peak of the UV-vis spectrum for the compounds: SWCNTs, HA, Ce6, and SWCNTs-HA-Ce6 at 200–800 nm. The maximum absorbance peak for SWCNTs was recorded at 268 nm [19]. There was no characteristic peak for biopolymer HA in the range of 200–800 nm [20]. The photosensitizer Ce6 dissolved in the solvent DMF, which acted as a blank for all the samples, recorded three characteristic peaks, one maximum absorption peak at 405 nm and two peaks at 503 nm and 664 nm [21] (Figure 1). The synthesised nanobiocomposite dispersed in Millipore water, which acted as a blank, and the major peaks of SWCNTs and Ce6 were observed in SWCNTs-HA-Ce6. These results indicated the successful synthesis of SWCNTs-HA-Ce6. UV-Vis spectroscopy was also used to determine the PS loading efficiency (PLE) for the synthesised nanobiocomposite. Due to the large surface area of SWCNTs, loading efficiency of Ce6 was achieved at 70%, calculated using the PLE formula (PLE (wt%) = (weight of loaded PS/weight of PS in feed) × 100%). The SWCNTs and Ce6 are hydrophobic in nature, while the synthesised nanobiocomposite showed excellent dispersibility due to the coupling of HA molecules. Dispersibility studies for all the samples were performed by dissolving 1 mg of SWCNTs and 1 mg of SWCNTs-HA-Ce6 with 1 mL of Millipore water separately and mixing it vigorously, and the image captured is shown in Figure S1. 

### 2.2. Fourier Transmittance of Infra-Red Spectroscopy of the Synthesised Nanobiocomposite

**Figure 2:**
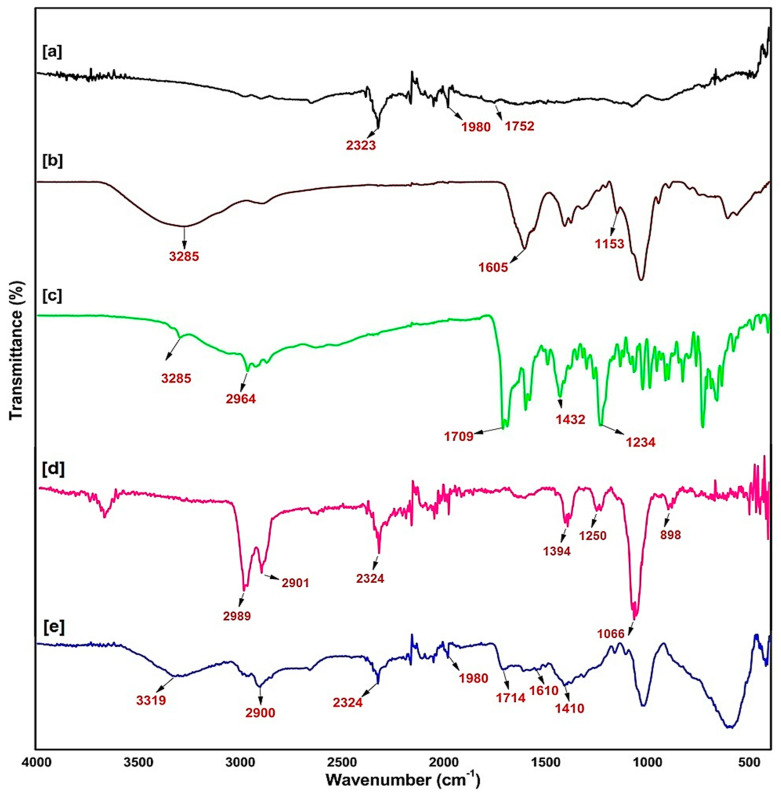
The Fourier transmittance infra-red spectrum of (**a**) SWCNTs, (**b**) HA, (**c**) Ce6, (**d**) SWCNTs-HA, (**e**) SWCNTs-HA-Ce6.

FTIR spectroscopy is an important and popular tool to study the surface chemistry (structural elucidation, bond shifting, and compound identifications) of the synthesised nanobiocomposite. The functional groups present in the compounds were identified based on the absorption frequency range. The SWCNTs, HA, Ce6 and SWCNTs-HA-Ce6 were analysed in FTIR and the appropriate peaks are recorded in Figure 2. The FTIR spectra of SWCNTs, depicted as a black line spectrum, showed a strong carbon absorption bond peak of C=C at 2323 cm^−1^, =C–H at 1980 cm^−1^, and carboxyl bond stretching C=O at 1752 cm^−1^ [22]. In the brown line spectra, the board 3285 cm^−1^ stretching represents the amide bond and medium peaks at 1605 cm^−1^ and 608 cm^−1^, the C–N minor stretching band observed at 1153 cm^−1^ [20] and 1040 cm^−1^ represents the functional group of COC of HA. The Ce6 spectrum depicted as a green line shows a strong stretching vibration peak of the carboxyl group at 1709 cm^−1^ [23], characteristics of O–H bands were observed at 3285 cm^−1^ and 1234 cm^−1^, and the band range of 2964 cm^−1^ and 1432 cm^−1^ indicates the C–H bond. The FTIR spectrum (pink line) of SWCNTs-HA illustrates peaks at 2323 cm^−1^ (C=C) that confirm the presence of basic functional units of a strong carbon absorption bond peak of SWCNTs, and the peaks at 1066 cm^−1^ confirm the presence of the COC functional group of HA [24]. The synthesised nanobiocomposite spectrum was depicted by the blue line; it showed the characteristic peaks of SWCNTs, HA and Ce6 with minor band shifts due to the functionalisation process. These results indicated the presence of the functional groups, the carboxyl and amide bonds spectrum vibrations present in the synthesised nanobiocomposite.

### 2.3. X-ray Diffraction (XRD) Analysis

**Figure 3:**
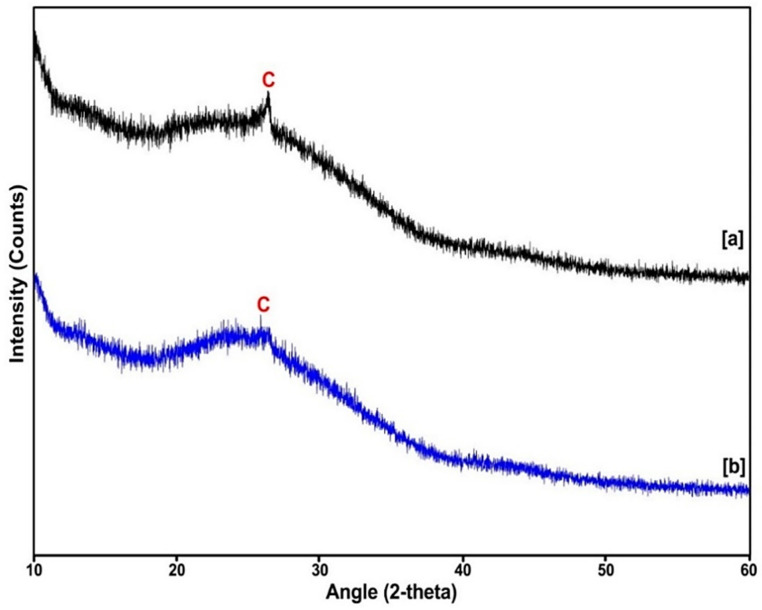
The XRD pattern of (**a**) SWCNTs and (**b**) SWCNTs-HA-Ce6.

The X-ray diffraction data confirms the crystallinity of the nanosized particles, as well as the structural properties of the synthesized nanobiocomposite by comparison to the Joint Committee on Powder Diffraction Standards (JCPDS) number. The SWCNTs and SWCNTs-HA-Ce6 were deposited on a crystal silicon wafer for analysis. The XRD patterns of the SWCNTs are shown in Figure 3 and indicate that the characteristic peak at 26.3° corresponds to C (002) planes, according to JCPDS # 751621 reflecting graphite. The plane 002 indicates the presence of SP2 bonded carbon arrangements of SWCNTs [25]. For the synthesised nanobiocomposite, there was a mild shift at 25.8° and the intensity of peak was less characteristic in the plane 002. This may be due to the coupling of HA and Ce6, and there were changes observed in the crystalline nature of the nanobiocomposite. These results confirm the addition of HA and Ce6 on the SWCNTs. 

### 2.4. Particle Size Distribution and Zeta Potential of the Synthesised Nanobiocomposite

**Figure 4:**
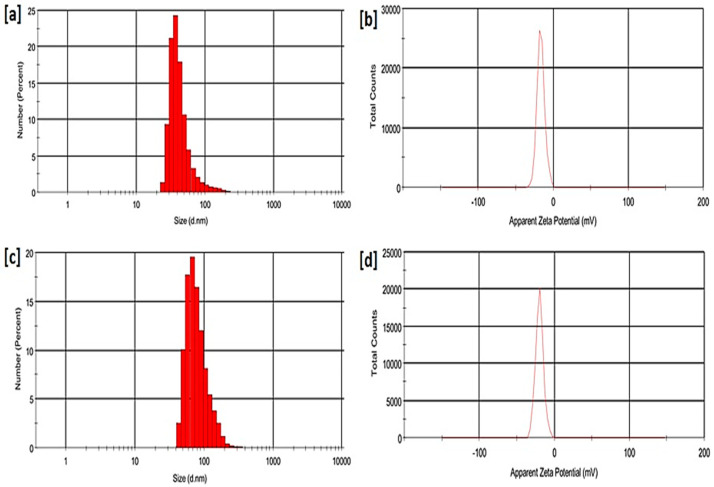
Dynamic laser scattering results: (**a**) particle size analysis of SWCNTs (**b**) Zeta potential of SWCNTs (**c**) particle size analysis of SWCNTsHA-Ce6 (**d**) Zeta potential of SWCNTs-HA-Ce6.

The zeta potential and particle size distribution were studied using the dynamic laser scattering technique (DLS) and the results are presented in Figure 4. Zeta potential measurements were used to characterise the surface charge and physical stability of the system. Particle size distribution was used to measure the size of the newly synthesised nanobiocomposite and in this study we compared the initial size of SWCNTs to the synthesised SWCNTs-HA-Ce6. The average size of the particles recorded was approximately 191 ± 4.6 nm for SWCNTs and 203 ± 6.6 nm for SWCNTs-HA-Ce6. The size of the nanobiocomposite increased due the coupling of HA and Ce6 molecules. The zeta potential analysis for SWCNTs was −17.8 ± 1.2 mV and for SWCNTs-HA-Ce6, it was −18.9 ± 1 mV. These results indicate that the SWCNTs and synthesized nanobiocomposite is stable. The stability of the nanoparticles is a crucial part of newly synthesised drug delivery systems, the zeta potential results strongly indicating the stability of SWCNTs-HA-Ce6.

### 2.5. Nanobiocomposite PDT Effects on Colon Cancer Cells

#### 2.5.1. Morphology Studies

**Figure 5:**
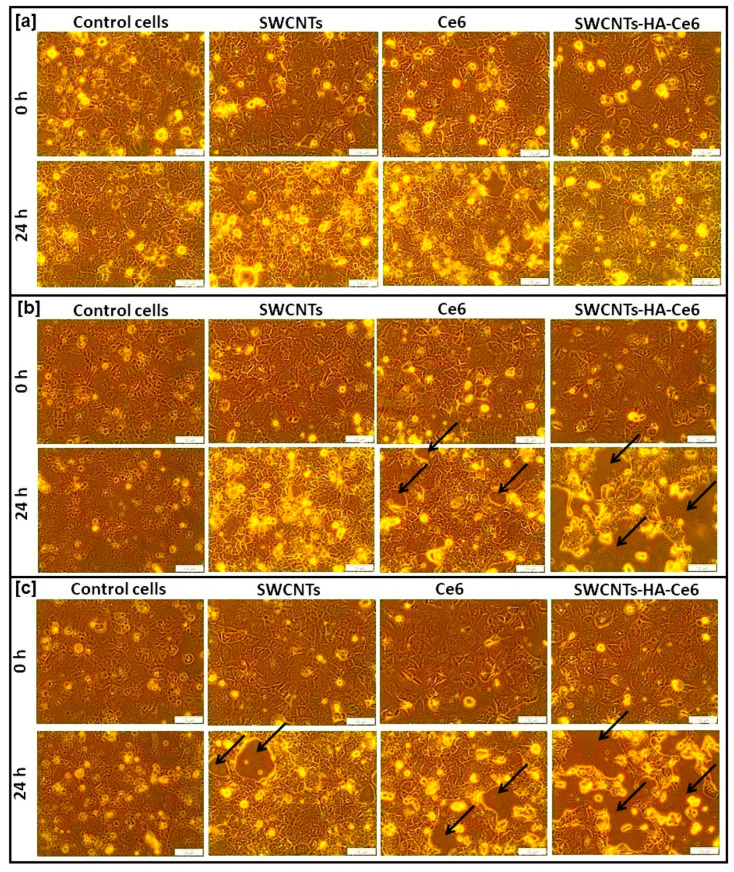
Microscopic images of untreated and treated cells. (**a**) group 1 cells (untreated) of 0 h and 24 h, (**b**) group 2 cells (5 J/cm^2^) of 0 h and 24 h (**c**) group 3 (10 J/cm^2^) cells of 0 h and 24 h. Scale bar represents 100 μm. Black arrow indicates the cellular death.

The morphological changes were observed through inverted microscopy. The Caco-2 cells were cultured to a population of 4 × 10^5^ cells on cell culture plates, and PDT treatment was performed. The microscopic images of all the samples were captured for 0 h and 24 h. Observing the image represented in Figure 5a revealed that there was full confluence of cells at 0 and 24 h, where cells received no irradiation but the treatment composites alone, whereas the cells receiving PDT treatment were damaged after 24 h Figure 5b,c.

#### 2.5.2. In Vitro Cytotoxicity Assay

**Figure 6:**
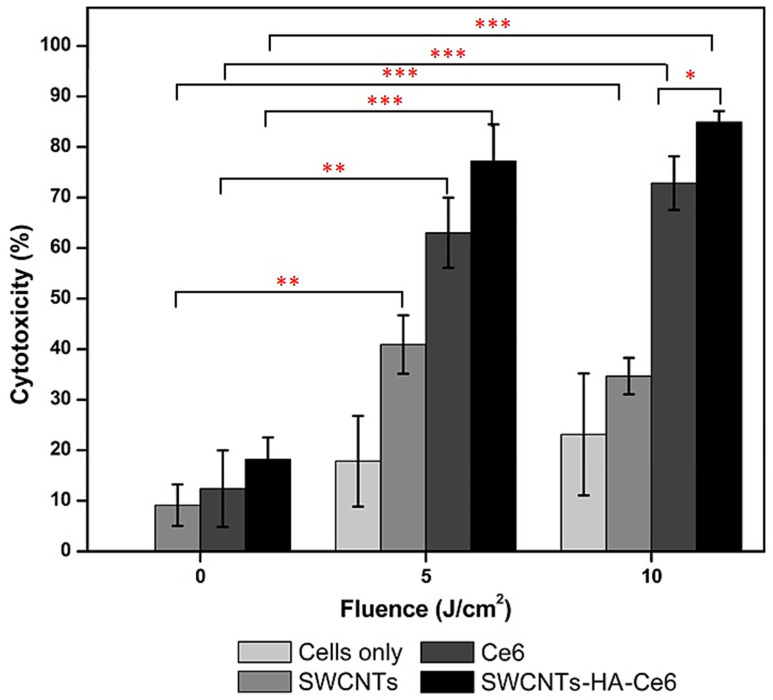
The cytotoxicity effects of SWCNTs, Ce6, SWCNTs-HA-Ce6 on Caco-2 cells determined by LDH assay. Significance is shown as * *p* < 0.05; ** *p* < 0.01; *** *p* < 0.001.

We performed the LDH cytotoxic assays for all the laser irradiated 24 h samples to evaluate the anticancer activity of SWCNTs, Ce6 and SWCNTs-HA-Ce6. A confluence of 0 J/cm^2^ was used throughout as control for this study. The LDH cytotoxic assay results are shown in Figure 6. Observation of the results revealed that the 0 J/cm^2^ (non-irradiated) cells showed a lower percentage of cytotoxic behavior for SWCNTs (9%), Ce6 (12%) and SWCNTs-HA-Ce6 (18%). The 10 J/cm^2^ irradiated cells showed significantly higher percentage of cell death for SWCNTs-HA-Ce6 (84.9%) compared to 5 J/cm^2^ irradiated cells (77%). More importantly, the present study also revealed that the synthesised nanobiocomposite showed an improvement in cell death percentage compared with free Ce6 treatment in both 5 J/cm^2^ (63%) and 10 J/cm^2^ (72.8%) irradiated cells. This could be a result of the enhanced bioavailability of Ce6 and their variations in uptake profile, which led to the increased cytotoxic behavior of SWCNTs-HA-Ce6 compared to free Ce6. The study also confirmed that the combined effect of laser irradiation (both 5 and 10 J/cm^2^) and nanobiocomposite treatment effectively inhibits the multiplication of Caco-2 cells. A comparison between 5 and 10 J/cm^2^ using the SWCNTs-HA-Ce6 was performed at 24 h, and even though an increase in cytotoxicity was seen, there was no statistically significant difference. Furthermore, we performed a comparison between Ce6 and SWCNTs-HA-Ce6 at both 5 and 10 J/cm^2^ and these results showed that no significant differences were observed at 5 J/cm^2^ (*p* = 0.118), but there was a significant increase in SWCNTs-HA-Ce6 compared to Ce6 in 10 J/cm^2^ (*p* = 0.041).

#### 2.5.3. Cell Death Analysis

**Figure 7:**
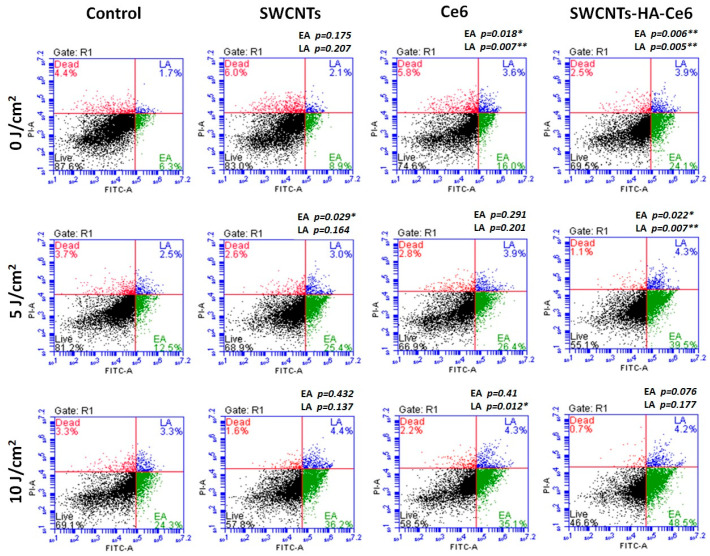
Flow cytometry Annexin V PI staining analysis of apoptosis.

The apoptotic profiles of SWCNTs, Ce6 and SWCNTs-HA-Ce6 on Caco-2 cells were evaluated using the Annexin V PI kit for flow cytometry after 24 h of incubation. Flow cytometry analysis of live (bottom left), early stage of apoptosis (bottom right), late stage of apoptosis (top right) and dead cells (top left) in the samples are represented in a four-quadrant image in Figure 7. The early and late stages of apoptosis from treated groups (SWCNTs, Ce6 and SWCNTs-HA-Ce6) were compared to the control and the *p*-values were calculated (shown in Figure 7).

In early stage apoptotic cells, a statistical comparison between control and SWCNTs, Ce6 and SWCNTs-HA-Ce6 was performed. The 0 J/cm^2^ treated samples results showed that there was a significant difference observed in Ce6 (*p* = 0.018) and SWCNTs-HA-Ce6 (*p* = 0.006), whereas there was no significant difference in SWCNTs (*p* = 0.175); the 5 J/cm^2^ treated samples results showed that there was a significant differences observed in SWCNTs (*p* = 0.029) and SWCNTs-HA-Ce6 (*p* = 0.022), whereas there was no significant differences in Ce6 (*p* = 0.291); and 10 J/cm^2^ treated samples results showed that there was no significant differences observed in SWCNTs (*p* = 0.432), Ce6 (*p* = 0.41) and SWCNTs-HA-Ce6 (*p* = 0.076). Similarly, a statistical comparison was performed between control and SWCNTs, Ce6 and SWCNTs-HA-Ce6 in late stage apoptotic cells and the 0 J/cm^2^ treated samples results showed that there was a significant differences observed in Ce6 (*p* = 0.007) and SWCNTs-HA-Ce6 (*p* = 0.005), whereas there was no significant differences in SWCNTs (*p* = 0.207); the 5 J/cm^2^ treated samples results showed that there was a significant difference observed in SWCNTs-HA-Ce6 (*p* = 0.007), whereas there was no significant difference in SWCNTs (*p* = 0.164) and Ce6 (*p* = 0.201); and 10 J/cm^2^ treated samples results showed that there was a significant difference observed in Ce6 (*p* = 0.012), whereas there was no significant difference in SWCNTs (*p* = 0.137) and SWCNTs-HA-Ce6 (*p* = 0.177).

## 3. Discussion

Currently, the rate of cancer patients and death cases is increasing rapidly due to modernized habits [26]. Researchers are focusing on treating cancer cells in different ways including chemotherapy, radiotherapy, multi-drug combinations, targeting, immunotherapy, photodynamic therapy, nanoparticle targeting [27], etc. Nanoparticles, such as metal-based nanoparticles, polymer-based nanoparticles, and carbon-based nanoparticles, may play an important role in cancer treatment and diagnosis [28,29]. Photodynamic therapy is a traditional and emerging treatment for cancer, focusing only on the cancerous cells without affecting the normal cells. A combination of PDT and nanotechnology-based treatments and diagnosis are novel and effective for cancer [30]. Carbon nanotubes were recently introduced as drug delivery systems due to the large surface area, unique physicochemical characteristics, uptake of cells and easy excretion from the body [31]. When the CNTs are rolled or coupled with biological substances such as proteins, blood compounds and polymers, the biocompatibility is increased, water dispersibility is also improved, and stability is maintained [32]. The SWCNTs are functionalised with a carboxyl group and the HA is activated with an amine group, both of which are chemically attached by a strong carbonyl bond. This biopolymer has improved the stability and water dispersibility of the SWCNTs [33]. It is very well known that electron rich molecules easily form π-π interactions with electron rich molecules and the bonds are very strong. Ce6 is hydrophobic in nature and is an electron rich molecule non-covalently coated with HA-SWCNTs for PDT with the purpose to treat cancer cells [34]. This synthesised nanobiocomposite was physically characterised to confirm the attachments of particles. The primary confirmation was done using UV-vis spectroscopy. The nanobiocomposite has both the Ce6 and SWCNTs peaks at respective wavelengths where the compound further undergoes the PDT effect. The FTIR results concluded that there is a shift between carboxyl and amine bonds when covalent functionalisation and non-covalent functionalisation of Ce6 show minor shifts between bonds and proper peaks, which are available on the nanobiocomposite. In the preparation of drug delivery systems, it is difficult to achieve stability and dispersibility and to maintain the size of the compounds to test the XRD characteristics, Zeta potential and particle size analysis (DLS). In the XRD pattern, SWCNTs result in the graphite form peak and SWCNTs-HA-Ce6 shows a slight shift of the peak, thus clearly indicating a change of the crystalline nature [35]. The size of the SWCNTs increased due to the coupling of HA and Ce6, then the zeta potential analysis clearly represents an improved stability of the synthesized particle in SWCNTs-HA-Ce6 (−18.9 ± 1 mV) compared to SWCNTs (−17.8 ± 1.2 mV). The PDT mainly works on laser irradiation at a specific wavelength which penetrates the body (biological tissues are more transparent) at the range of 600–800 nm [29]. Ce6, which is a naturally derived molecule activated by the light source in the range of 660–665 nm, is our selected PS molecule [36]. Xie’s group developed a photostable long blood circulating SWCNTs system to deliver Ce6 and achieved an enhanced tumor accumulation through synergistic tumor therapy using PDT/PTT [37]. Xiao et al. (2012) reported improved solubility of Ce6 (hydrophobic in nature) and performed laser irradiation studies in HeLa cells using 20 J [21]. Herein, we treated cancer cells using PDT in two different fluences of 5 and 10 J/cm^2^ at the wavelength of 660 nm. IC50 concentration of compounds from cytotoxicity studies were selected for PDT experiments. We used HA to improve the solubility of SWCNTs and the loading of Ce6 because it has been reported that HA is considered a proven compound to improve the solubility and multifunctionality of SWCNTs [38]. A HA-based graphene sheet nanomaterial for the delivery and an effective cellular internalization of Ce6 has been shown to be effective [39]. Further, the PDT treatment results showed a higher level of cytotoxicity using the synthesized nanobiocomposite compared to Ce6 alone. In cell death analysis (flow cytometry), results indicated that the combined effect of the nanobiocomposite and PDT (both 5 and 10 J/cm^2^ laser irradiated) plays a significant role in enhancing the cell counts in both early and late stage apoptosis compared to their controls. A close observation of this study revealed that there were increased cell counts in early stage apoptosis in 10 J/cm^2^ (41.9 ± 6.65) compared to 5 J/cm^2^ (36 ± 6.4). Similarly, we observed the results of live cell counts and it showed a decreased percentage in 10 J/cm^2^ (53.4 ± 6.8) compared to 5 J/cm^2^ (58.27 ± 5.9). Thus, SWCNTs-HA-Ce6 and 10 J/cm^2^ laser irradiation showed a better apoptotic activity than free Ce6 and empty SWCNTs.

The nanobiocomposite systems based on SWCNTs exhibit an excellent PS delivery due to the large surface area and strong bonding with a different choice of PS molecules [40]. Overall, the present study confirmed that SWCNTs-HA-Ce6 and PDT enhanced apoptosis of CRC through flow cytometry results. Future recommendations for the cell death analysis of nanobiocomposites (SWCNTs-HA-Ce6) include TUNEL assay, caspase family and bcl-2 expression through western blotting studies to further validate the claims of the SWCNTs-HA-Ce6 in relation to the cell death mechanisms.

## 4. Materials and Methods

### 4.1. Experiment

Carboxylated SWCNTs were purchased from Sigma Aldrich (St. Louis, MO, USA), and hyaluronic acid was obtained from Sigma. The photosensitizer Chlorin e6 was obtained from Santa Cruz Biotechnology (Dallas, TX, USA). N-ethylcarbodiimde hydrochloride (EDC.HCl), N-hydroxy succinimide (NHS), and dimethyl sulfoxide (DMF) were purchased from Sigma Aldrich. Caucasian colon adenocarcinoma Caco-2 cells human cell lines were obtained from ATCC. The cell culture growth media was Dulbecco’s modified Eagle’s medium (DMEM) obtained from Sigma Aldrich. CytoTox 96^®^ Non-Radioactive cytotoxicity assay was obtained from Promega Corporation (Madison, WI, USA). For the flow cytometry, FITC Annexin V Apoptosis detection kit I was from BD Pharmingen (San Jose, CA, USA).

### 4.2. Synthesis of the Nanobiocomposite 

The purchased SWCNTs were refluxed with nitric acid (HNO_3_) for 24 h to eliminate the trace elements and to make a purified form. They were then filtered through a mixed cellulose ester filtration membrane pore size of 0.22 μm. Hyaluronic acid was added into the formamide to dissolve it. EDC.HCl and NHS were then added to the HA-formamide solution followed by stirring for 30 min. Trimethylamine (1 mL) was added drop by drop and kept cool using an ice bath, and then the solution was stirred and kept at room temperature (RT) for 2 h, which activated the HA. The reacted product was collected in membrane filter through rinsing repeatedly with acetone. We dialysed the activated HA for 48 h to remove acetone and unreacted EDC.HCl and NHS. The SWCNTs-COOH and HA-NH_2_ were added into formamide, sonicated for 10 min, EDC.HCl and NHS were added, and then it was stirred at RT for 15 min. After that, 180 μL of triethylamine was added drop by drop in the ice bath and reacted at RT for 24 h. The reaction solution was cooled with excess precooled acetone and centrifuged at 10,000 rpm for 15 min. Finally, the precipitation was dissolved with water and dialyzed by a dialysis bag for 48 h to remove free HA and the synthesised products [12]. The non-covalent binding of Ce6 on the walls of SWCNTs was achieved by the π-π interaction technique. Ce6 and HA-SWCNTs were dissolved in N, N-dimethyl formamide (DMF). The solution was then sonicated for 10 min. The mixture was stirred overnight and kept at RT. The final product was filtered using membrane filters (0.22 μm) and rinsed with DMF solution to remove unloaded Ce6. The final solution was then rinsed with milli-Q water and acetone to remove residual DMF [13]. The amount of unbound Ce6 was measured using UV-vis spectroscopy of the maximum absorbance peak at 405 nm for determination of drug loading efficiency (PLE).

### 4.3. Spectrum Analysis of Synthesised the Nanobiocomposite

Ultraviolet visible (UV-vis) spectroscopy was used to record the maximum absorbance peak at specific wavelengths based on the particle characteristics. The spectral analysis was performed using a UV-Vis Spectroscopy (Genova 7315, Life Science Spectrophotometer, JENWAY, Staffordshire, UK) at a resolution of 1 nm from 200 to 800 nm. Milli-Q water was used as a blank for all the compounds in quartz cuvette.

### 4.4. Fourier Transform Electron Microscopy (FTIR) of the Nanobiocomposite

The FTIR spectroscopy was used for the analysis of the bonds and functional groups present and shifting. Moreover, potassium bromide (KBr) was used as well as the Hydraulic Pellet Press method to produce a thin sample of KBr pellet that was then subjected to FTIR analysis. The compounds were added on the surface of pelleted KBr to measure FTIR spectroscopy. The frequency range was measured as wave numbers, typically over the range 4000–500 cm^−1^ using the JASCO FT/IR-6300 Series, Japan.

### 4.5. X-ray Diffraction (XRD) of the Nanobiocomposite

X-ray diffraction measures structures of crystalline compounds and surface chemistry. The XRD patterns were determined using a diffractometer equipped with a rotating target X-ray tube and a wide-angle goniometer. The X-ray source were Kα radiated from a copper target with a graphite monochromator. The X-ray tube was operated at a potential of 45 kV and a current of 40 mA. The range (2 θ) of scans was performed from 10° to 80° at a speed of 4° per min at increments of 0.05°. The samples were analysed using the Rigaku Ultima lV instrument and the data were collected from PDXL analysis software (Tokyo, Japan).

### 4.6. Particle Size and Zeta Potential (DLS Method) of Nanobiocomposite

The synthesised nanobiocomposite size distribution (hydrodynamic diameter) and Zeta potential were studied by the dynamic light scattering (DLS) method (Zeta sizer ver. 7.10, Malvern Instruments Ltd., Malvern, UK). The samples were diluted using Millipore water, measurements were carried out using disposable cuvettes (DT S0012) in triplicate. For the light method, samples were measured at a fixed angle of 90° at 25 °C.

### 4.7. Cell Culture

The human epithelial colorectal adenocarcinoma cell line purchased from ATCC was used in this study (Caco-2, ATCC^®^ HTB-37TM, Manassas, VA, USA). The colon cancer cell line was maintained in complete growth media of Dulbecco’s modified Eagle medium (DMEM) supplemented with 10% fetal bovine serum (Biochrom, S0615), 2 mM L-glutamine, 0.5% penicillin/streptomycin (Sigma, P4333) and 0.5% amphotericin B (Sigma, A2942). Cultured cells were incubated at 37 °C in 5% CO_2_ and 85% humidity.

### 4.8. Photodynamic Effects (PDT) 

The red-light lasers were obtained from the National Laser Centre of South Africa. The laser output was measured using a FeildMate Laser Power Meter (FieldMate, Coherent, Power sens detector) and the laser parameters are summarized in Table 1. The cells were cultured in a 3.4 cm petri dish where the laser spot size covers the monolayer of cells. The height of the light source was 8 cm above the cells. To treat the cancer cells, red light lasers were used at the wavelength of 660 nm, which is the excited range of the Ce6 photosensitizer. The cells were divided into three groups: group 1: cells alone and cells with SWCNTs, Ce6 and SWCNTs-HA-Ce6 without treatment; group 2: cells alone and cells with SWCNTs, Ce6 and SWCNTs-HA-Ce6 with treatment of fluence 5 J/cm^2^; group 3: cells alone and cells with SWCNTs, Ce6 and SWCNTs-HA-Ce6 with treatment of fluence 10 J/cm^2^. An IC50 established at 2.56 μg/mL for the nanobiocomposite was used throughout this study.

#### 4.8.1. Morphology

The live and dead cell morphology before and after irradiation was viewed under a CKX41 inverted light microscope (Olympus, Wirsam) connected to a camera with analysis getIT software.

#### 4.8.2. Cytotoxicity

The cytotoxicity level of the samples was estimated using Cyto Tox 96^®^ Non-Radioactive cytotoxicity assay. The CytoTox 96^®^ assay quantitatively measures the lactate dehydrogenase (LDH), which was released from cells during lysis. Fifty microliters of control and test samples were added in a 96 well plate and the same amount of LDH reagent was added in the wells, then incubated in the dark at room temperature for 30 min. The colorimetric compound was measured spectrophotometrically at 490 nm using a multilabel Counter (Perkin Elmer, VICTOR3™ Multilabel Plate Reader, 1420).

#### 4.8.3. Cell Death

The assay uses Annexin V–fluorescein isothiocyanate (FITC) and propidium iodide (PI) (BD Biosciences, 556547, Gauteng, South Africa) to identify phosphatidyl serine sites on the membrane of apoptotic cells, as well as sites of membrane damage in necrotic cells, respectively. The assay was done according to the manufacturer’s instructions and analysed on the BD Accuri™ C6 flow cytometer. Annexin V–FITC was detected as green fluorescence and PI as red. After treatment and incubation, the cells from all groups were detached and washed with Hanks Balanced Salt Solution (H9394, Sigma-Aldrich, South Africa). Cells were resuspended in the 1X binding buffer at a concentration of 10^6^ cells/mL, and 100 μL of the cell suspension was transferred into flow cytometry tubes. Five microliters each of the Annexin V–FITC and PI reagents were added, and the tubes were thoroughly mixed and incubated for 10 min at RT in the dark. Flow cytometric analysis was performed within one hour at a rate of 400 events per second with a limit of 350 μL for each sample. Data acquisition proceeded until 10,000 events were obtained for every sample. Data was analysed on the BD C Sampler software.

### 4.9. Statistical Analysis

The results were accumulated, processed for graphing, and data analysis was done with Origin Pro 8 SRO (v8.0724) and BD CSampler™ software. All experiments were done in triplicate to monitor for the reproducibility of the results, and all data is expressed as the mean ± standard deviation. Difference between groups were determined using the one-tailed student *t*-test.

## Figures and Tables

**Table 1:** Laser parameters for photodynamic therapy.

PARAMETERS	
Laser type semiconductor	Diode
Wavelength (nm)	660
Wave emission	Continuous
Fluences (J/cm^2^)	5 and 10
Irradiation time	7 min 16s 14 min 32 s
Photosensitizer	Chlorin e6

## Supplementary Materials

Supplementary materials can be found at https://www.mdpi.com/1422-0067/21/13/4745/s1.

## Abbreviations

SWCNTs	Single walled carbon nanotubes
HA	Hyaluronic acid
Ce6	Chlorin e6
PDT	Photodynamic therapy
PS	Photosensitizers
DMF	Dimethylsulfoxide
EDC.HCl	N-ethylcarbodiimde hydrochloride
NHS	N-hydroxysuccinimide
UV	Ultraviolet
FTIR	Fourier transform infra-red
XRD	X-ray diffraction
DLS	Dynamic laser scattering
PI	Propidium idode
DMEM	Dulbecco’s Modified Eagle Medium
°C	Degree Celsius
μL	Microlitre
mL	Millilitre
mA	Milli ampere
kV	Kilo voltage
J	Joules
h	Hours
min	Minutes
s	Seconds
rpm	Rotation peer minute
nm	Nanometer
mV	Milli voltage
cm	Centimetre
RT	Room temperature
