# Supplementary material for: Effective Photodynamic Therapy for Colon Cancer Cells Using Chlorin e6 Coated Hyaluronic Acid-Based Carbon Nanotubes
Source: Int J Mol Sci. 2020 Jul 3;21(13):4745. doi: 10.3390/ijms21134745 (PMC7369763; doi:10.3390/ijms21134745)
Supplement: Supplementary file 1 [file ijms-21-04745-s001.pdf]

Supplementary information

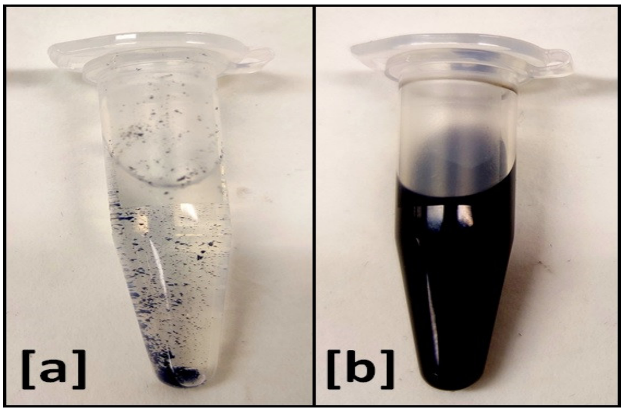

Figure S1. [a] SWCNTs and [b] SWCNTs-HA-Ce6. The dispersion was diluted at a concentration of 1mg/ml at room temperature.
